# Formation of Water-Channel by Propylene Glycol into Polymer for Porous Materials

**DOI:** 10.3390/membranes11110881

**Published:** 2021-11-16

**Authors:** Seong Ho Hong, Younghyun Cho, Sang Wook Kang

**Affiliations:** 1 Department of Chemistry, Sangmyung University, Seoul 03016, Korea; wkn7836@daum.net; 2 Department of Energy Systems Engineering, Soonchunhyang University, Asan 31538, Korea; 3 Department of Chemistry and Energy Engineering, Sangmyung University, Seoul 03016, Korea

**Keywords:** cellulose acetate, water pressure, pore generation, propylene glycol, hydration region, hydrophilic functional group

## Abstract

In this study, a porous membrane with a cellulose acetate (CA) matrix was fabricated using propylene glycol with a water pressure treatment without a metal salt as an additive. The water pressure treatment of the fabricated CA membrane with propylene glycol yielded nanopores. The nanopores were formed as the additives in the CA chains led to plasticization. The weakened chains of the parts where the plasticization occurred were broken by the water pressure, which generated the pores. Compared to the previous study with glycerin as an additive, the size of the hydration region was controlled by the number of hydrophilic functional groups. When water pressure was applied to the CA membrane containing propylene glycol as an additive, the hydration area was small, so it was effective to control the pore size and the number of nano pores than glycerin. In addition, the number of nanopores and pore size could be easily adjusted by the water pressure. The porosity of the membrane was increased owing to the trace amount of propylene glycol, confirmed by scanning electron microscopy (SEM) and porosimetry. The interaction between the CA and propylene glycol was verified by Fourier-transform infrared spectroscopy (FT-IR) and thermogravimetric analysis (TGA). Consequently, it was the optimum composition to generate pores at the CA/propylene glycol 1:0.2 ratio, and porosity of 69.7% and average pore diameter of 300 nm was confirmed. Since it is a membrane with high porosity and nano sized pores, it is expected to be applied in various fields.

## 1. Introduction

Porous materials are used in various applications, including batteries, separators, medicine applications, and organic syntheses [1,2,3,4,5,6,7,8]. They have been mainly used as water-treatment membranes, gas-separation membranes, battery separators, and catalysts of various syntheses [9,10,11,12,13,14,15,16]. 

In a recent study, Huang et al. have used a porous membrane to resist the fouling effect. The porous membrane was coated with a lithium exchange vermiculite to prevent contamination and used for oil–water separation [17]. Plasticization or cross-linking of additives in a polymer and generation of pores by applying a physical force have been used in the manufacturing of a porous membrane [18]. The porosity of the polymer structure has been controlled by changing the number or type of multi-functional groups under catalyst control during the polymer synthesis [19]. In the field of battery separators, Xu et al. have reported that lithium–sulfur batteries exhibit dendrite growth in the lithium metal anodes owing to the dissolution and diffusion of polysulfides [20]. This has been overcome by assembling a two-dimensional peeled vermiculite sheet, suppressing the diffusion of polysulfides across the separator through electrostatic interactions and steric hindrance. In the field of porous catalysts, Raso et al. have reported that zeolite membrane reactors can be used for hydrogenation of CO_2_ to methanol. The removal of water during the reaction increases the reaction rate and achievable conversion rate. In their study, zeolite has achieved the highest H_2_O/H_2_ separation performance [21]. Sun et al. have reported that the advent of porous organic polymers (POPs) enables novel applications of heterogeneous catalysts owing to their unique structural characteristics. Representative recent developments of POP-based catalysts with hierarchical porous structures have been presented [22]. Sun et al. have reported a hydroxyapatite/chitosan (HAP/CS) porous material having interconnected three-dimensional macro pores with sizes of 100–300 mm. The adsorption capacity of the HAP/CS porous material for Pb(II) ions under flow conditions was 264.42 mg/g, while that of a CS porous material was 5.67 mg/g.

Therefore, the HAP/CS porous material has a higher potential to remove Pb(II) ions from aqueous solutions. The better adsorption properties of the HAP/CS porous material than those of the CS porous material are attributed to the HAP particles in the composite material [23]. 

Water treatment method was proposed, since the existing porous material manufacturing process has a problem that there are many steps and is expensive. In recent studies, our group have enhanced porous films through an efficient pore-generation method with small-molecular-weight organic additives and water pressure based on a cellulose acetate (CA) polymer, which is cheap, thermally stable, and environmentally friendly [24,25,26]. Our group proposed a method for the generation of pores by using metal salts, such as magnesium nitrate, nickel (II) nitrate, and zinc (II) nitrate, as additives [27,28]. This method has been used to form pores applying a water-pressure treatment on the portion where hydrated metal ion and nitrate ion are present in the CA film because nitrate ion has good hydration effect. However, the use of the previous method was not effective, as the metal salts as additives are expensive. In the first metal-free pore generation research, glycerin was substituted for metal salts. In the pore-generation experiment in the previous study where glycerin was used as an additive, the hydration size of the glycerin has increased owing to the numerous hydrophilic groups causing plasticization in the CA chains [29]. In this study, propylene glycol, a derivative of glycerin, a byproduct of the process, with a low cost is used owing to the different number of hydroxyl groups. Propylene glycol has a lower hydrophilicity than that of glycerin. The propylene glycol is highly hydrated by the hydrophilic functional hydroxyl groups. In addition, plasticization occurs in a smaller area than that for glycerin. Thus, the pore size and porosity can be easily controlled by the number of hydrophilic groups. Considering the hydration by two hydroxyl groups per propylene glycol molecule, the flexibility is expected to increase owing to the plasticization effect in the CA matrix. The porosity and pores generated upon the water pressure treatment were confirmed by scanning electron microscopy (SEM) (JSM-5600LV, JEOL, Tokyo, Japan) and porosimetry (MicroActive AutoPore V 9600, Micrometrics, Norcross, GA, USA) [30]. Fourier-transform infrared (FTIR) spectroscopy (VERTEX 70/70V FT-IR spectrometers, Bruker Optics, Billerica, MA, USA) and thermogravimetric analysis (TGA) (Universal V4. 5A, TA instruments, New Castle, DE, USA) were carried out to confirm the dispersion in the membrane.

## 2. Materials and Methods

### 2.1. Materials

CA (Mn ~ 30,000 g/mol) was purchased from Aldrich Co. Propylene glycol (purity 99.0%) and acetone (purity 99.8%) were purchased from Daejung Chemical & Metals. All reagents were used without pretreatment.

### 2.2. Methods

#### 2.2.1. Membrane Fabrication & Water Pressure Applying Membrane

A 10% (wt/wt) CA (Mn ~ 30,000) solution was prepared by dissolving CA in acetone/water (8:2 wt/wt) by weight ratio, with propylene glycol with a CA/propylene glycol (1:0.2) by molar ratio. Additives, CA, water, and acetone were then added, in this order. The solution was stirred for 4 h. When preparing the solution, add propylene glycol, cellulose acetate, water, and acetone, in the order. Subsequently, the solution was cast on a glass plate to a thickness of 200 µm using a Baker applicator, and then dried for 20 min in a thermo-hygrostat at 25 °C and humidity of 50%. The dried film was using a water-treatment equipment, which can be applying water pressure to fabricated film from 2 bar to 8 bar. The sizes of the various pores formed in the membrane were measured through the water flow rates in a unit of L/m^2^h (LMH).

#### 2.2.2. Water Treatment Equipment

**Scheme 1:**
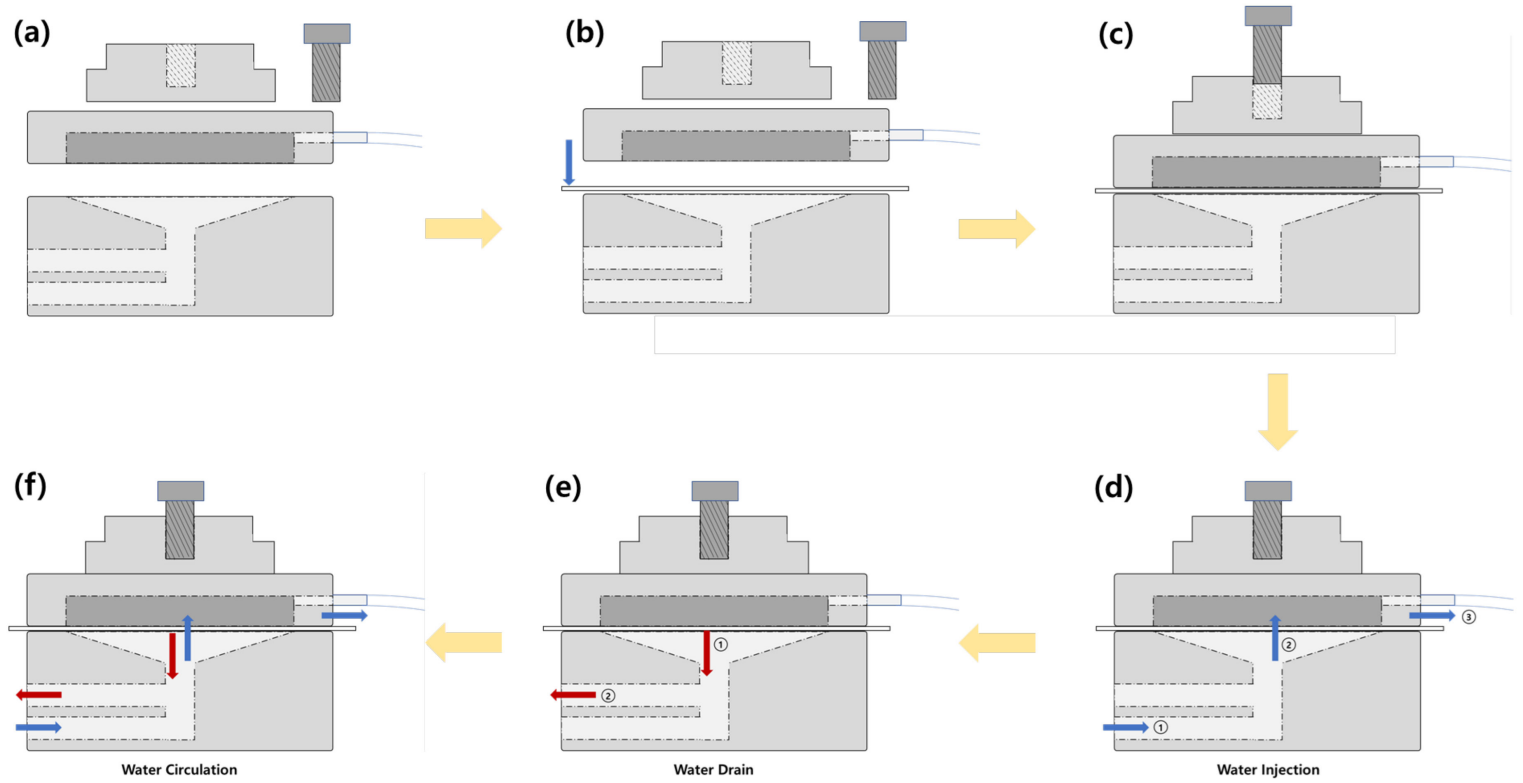
Schematic diagram of water treatment equipment. (**a**) equipment parts, (**b**), (**c**) membrane installation, (**d**) equipment water injection, (**e**) equipment water drain, (**f**) overall water cycle

Scheme 1a is a diagram of a device that applies water pressure to a fabricated membrane. This equipment was manufactured in our laboratory. In Scheme 1b,c, lock the equipment using the screws after placing the prepared membrane on the equipment in order of the schematic. Then, when water is injected, the water pressure of the membrane is applied and the water that has passed while piercing the membrane comes out through the route 3 in the water injection figure at Scheme 1d. Water pressure is controlled using the flow rate controller and the rpm of the motor used when water is injected. The flux data are measured using the flow velocity of water coming out of the membrane at 2 to 8 bar. Water that has not passed through the film will drain along the path of the water drain shown in the Scheme 1e. As a result, this equipment is used to circulate water injection and drainage, applying continuous water pressure to the membrane in Scheme 1f. With this device, water pressure can be applied to a certain circular area, so the value obtained by distributing the volume of water that passes through this effective area and comes out per hour to the size of this effective area is flux.

#### 2.2.3. SEM Sampling

The film made through water pressure treatment is dried enough to blow away moisture. When observing the surface of the membrane, cut the area to be observed and attach it to the top of the grid, and when observing the cross-section, freeze the film in liquid nitrogen to prevent tension from being applied to the film, break it, and attach it to the side of the grid.

#### 2.2.4. FTIR Sampling

The membrane after applying water pressure and the membrane before applying water pressure are dried in vacuum oven for 3 days to remove residual solvent. For comparison, a neat CA film is prepared and vacuum dried for 3 days without applying water pressure. Then, the film is cut into small portions and subjected to FTIR analysis.

#### 2.2.5. TGA Sampling

The membrane before and after applying water pressure are dried in vacuum oven for 3 days to remove residual solvent. For comparison, a neat CA film is fabricated and vacuum dried for 3 days without applying water pressure. Cut the dried films into small pieces, place about 20~30 mg on the platinum tray, and heat it from room temperature to 800 degrees.

## 3. Results and Discussion

### 3.1. Scanning Electron Microscope (SEM)

**Figure 1:**
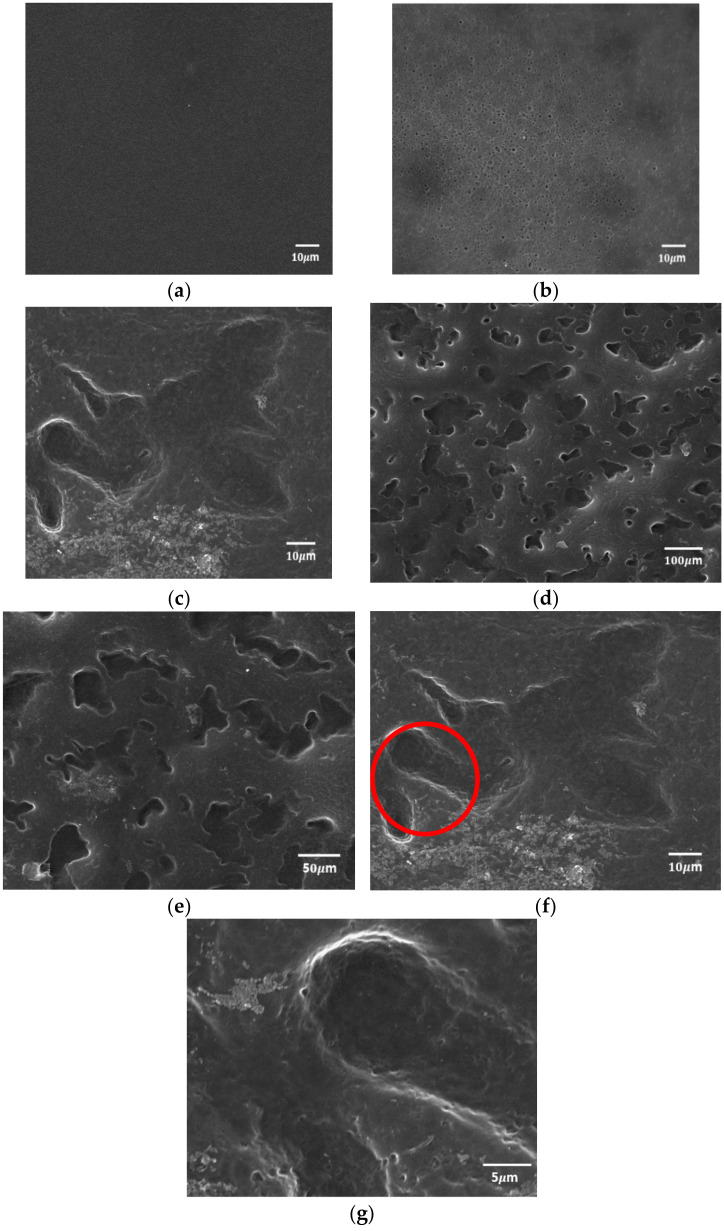
Scanning electron microscopy (SEM) images of the (**a**) neat cellulose acetate (CA), (**b**) membrane fabricated using the 1:0.2 CA/propylene glycol dissolved in acetone/water (8:2 wt/wt) before the water pressure treatment, (**c**) membrane fabricated using the 1:0.2 CA/propylene glycol dissolved in acetone/water (8:2 wt/wt) after the water pressure treatment (8 bar), the membrane fabricated using the 1:0.2 CA/propylene glycol dissolved in acetone/water (8:2 wt/wt) after applying the water pressure treatment (8 bar), (**d**) magnifications: ×140 and (**e**) magnifications: ×300. Magnified images of the areas within the red circles in (**e**) is (**f**) and red in (**f**) is (**g**).

SEM was applied to confirm the morphology and size of the pores generated on the membrane. Figure 1a shows the neat CA, Figure 1b shows the film fabricated using CA/propylene glycol (1:0.2) dissolved in acetone/water (8:2 wt/wt), which was not water-pressure-treated, and Figure 1c shows the membrane fabricated using the CA/propylene glycol (1:0.2) dissolved in acetone/water (8:2 wt/wt), which was treated by a water pressure of 8 bar. As can be seen in Figure 1a, there were no pores on the surface of the pure CA film. The membrane in Figure 1b had pores generated by the solvent evaporation. The film in Figure 1c has various pores generated by hydraulic pressure treatment (8 bar). Therefore, it was confirmed that the plasticized CA matrix, and when water pressure was applied to this part, pores were easily formed in the weakened part by plasticization.

Figure 1d–f shows the magnified image of the membrane (1:0.2 CA/propylene glycol dissolved in acetone/water (8:2 wt/wt)) after the water pressure treatment (8 bar) and the area indicated by the red circle. The pores of the CA membrane were successfully created by the plasticized region, and a pore could be easily generated through water pressure in the weakened area due to plasticization. As the molecular weight of CA was small, when the cross-section of the membrane was investigated or when the surface was magnified 3600 times (or more), the cross-section and surface could not be observed as the polymer collapsed during the SEM imaging.

### 3.2. Fourier Transform Infrared (FTIR)

**Figure 2:**
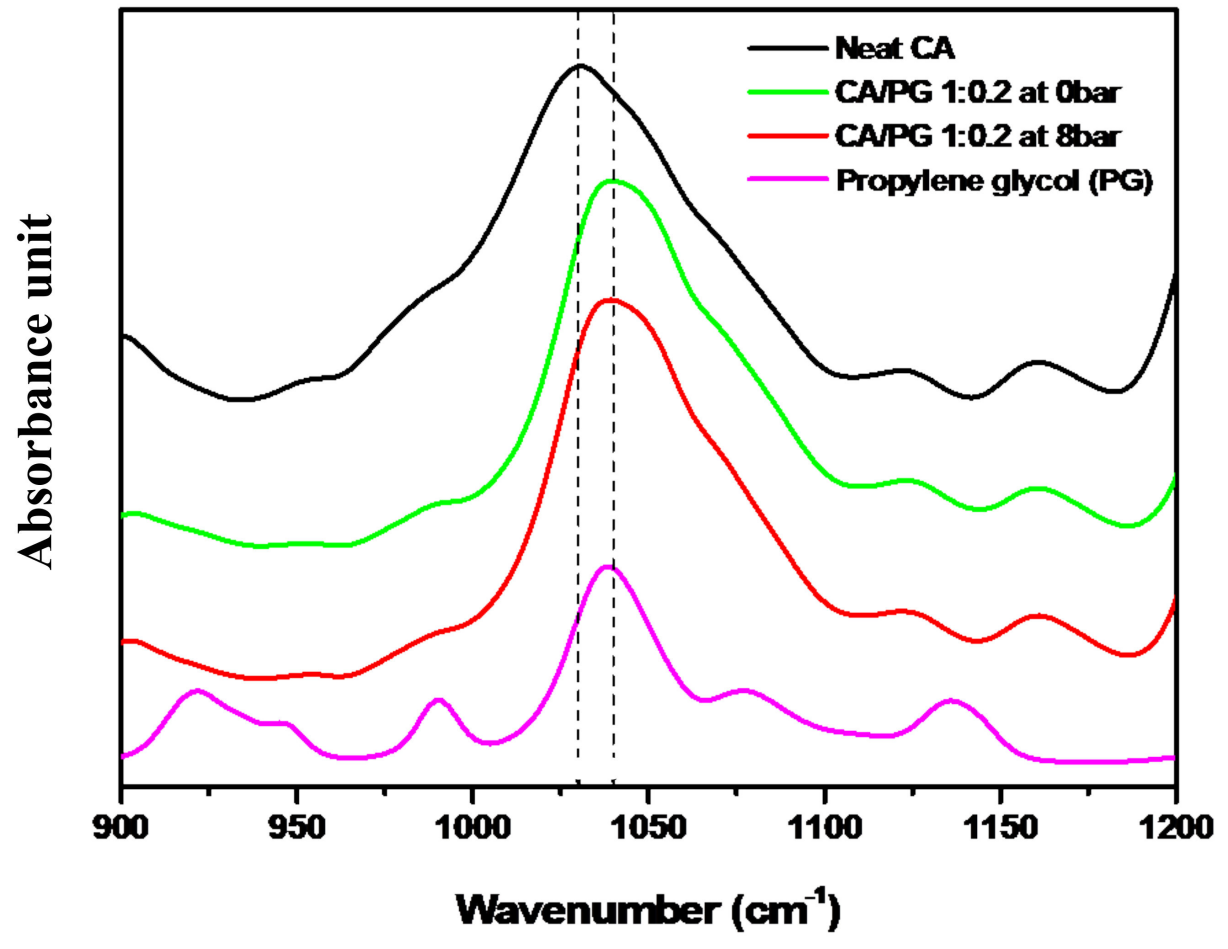
FTIR spectra showing the ether group of neat CA, 1:0.2 CA/propylene glycol (0 bar, 8 bar) and propylene glycol.

The interaction of the CA and propylene glycol was analyzed by FTIR spectroscopy. Figure 2 shows the ether group peaks of the neat CA at 1031 cm^−1^, 1:0.2 CA/propylene glycol (0 bar) at 1039 cm^−1^ and 1:0.2 CA/propylene glycol (8 bar) at 1039 cm^−1^. When the propylene glycol was added as an additive at pure CA, the ether peak was shifted from 1031 to 1039 cm^−1^. The ether peak of the CA was lower than the common ether peak, such as diethyl ether and diphenyl ether, due to the strong intermolecular interaction between the CA chains, a polymer with low molecular weight CA. The intermolecular interactions between the CA chains in the polymer weakened, when the even dispersion of the propylene glycol into the polymer. Thus, the dispersed propylene glycol plasticized the CA polymer chains.

As the strong intermolecular interactions in the CA disappeared, the blue-shift occurred in the wavenumber of cellulose acetate ether peak. The peak was observed at 1039 cm^−1^ after the water pressure treatment (8 bar), which indicates that the propylene glycol was dispersed and maintained in the matrix portion where pores were not generated even after applying water pressure.

**Figure 3:**
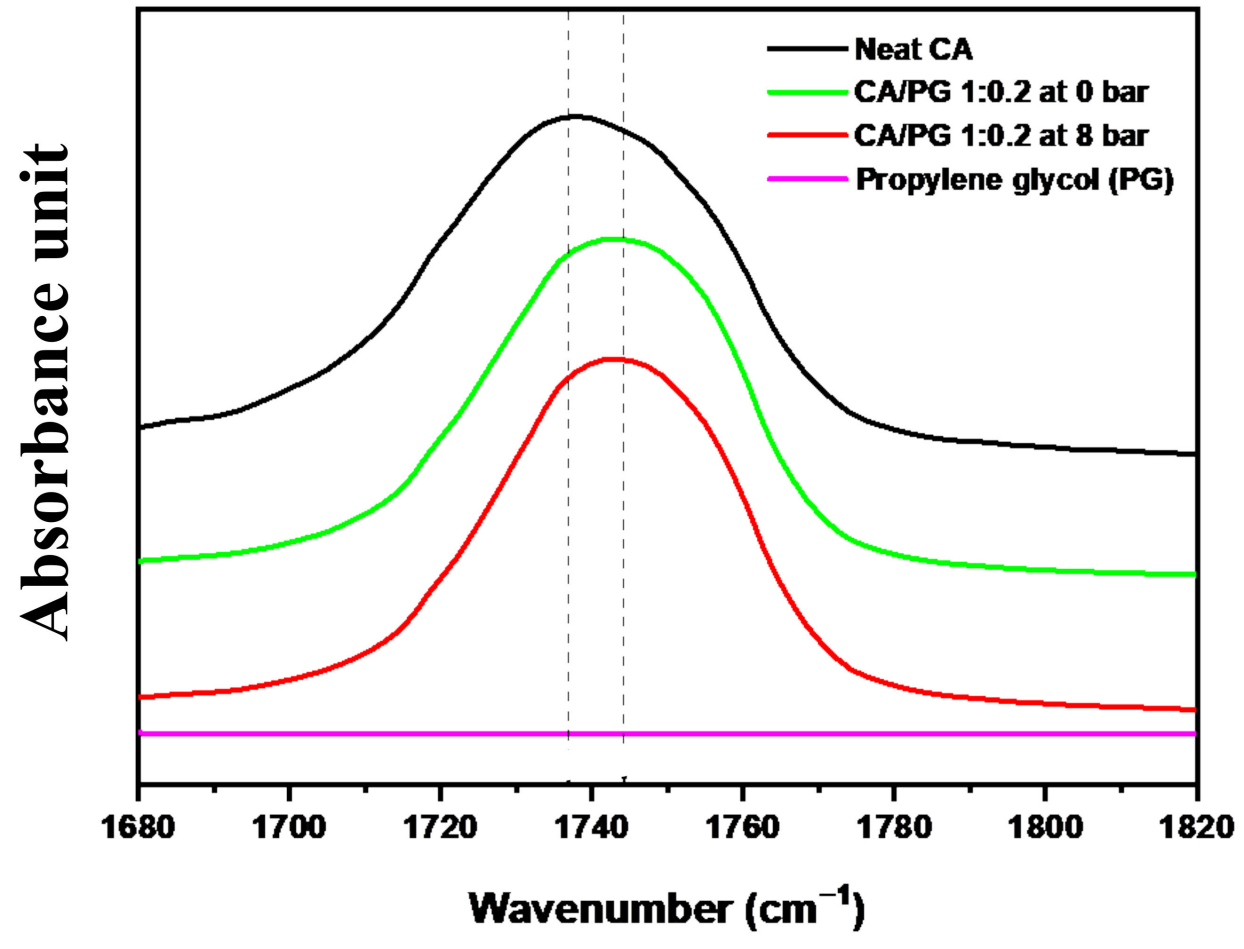
FTIR spectra showing the carbonyl group peaks of the neat CA, 1:0.2 CA/propylene glycol (0 bar, 8 bar) and propylene glycol.

Figure 3 shows carbonyl peaks of the neat CA at 1737 cm^−1^ and 1:0.2 CA/propylene glycol (0 bar, 8 bar) at 1745 cm^−1^. When the propylene glycol was added as an additive, the CA matrix was affected by the plasticization and the carbonyl peak was shifted from 1737 to 1745 cm^−1^. Upon the even dispersion of the propylene glycol in the polymer (small-molecular-weight CA chains with strong intermolecular interactions), the intermolecular interactions between the CA chains in the polymer weakened. The dispersed propylene glycol led to plasticization in the CA polymer. As the strong intermolecular interactions in the CA weakened, the blue-shift occurred in the wavenumber of cellulose acetate carbonyl peak. Although there is no carbonyl peak in propylene glycol, compared to neat CA, the state of propylene glycol is blue-shift, so propylene glycol has an interaction with the carbonyl group of CA. As the peak observed at 1745 cm^−1^ even after hydrostatic treatment (8 bar), propylene glycol was dispersed and remained in the part of the matrix where pores were not generated.

### 3.3. TG Analysis

**Figure 4:**
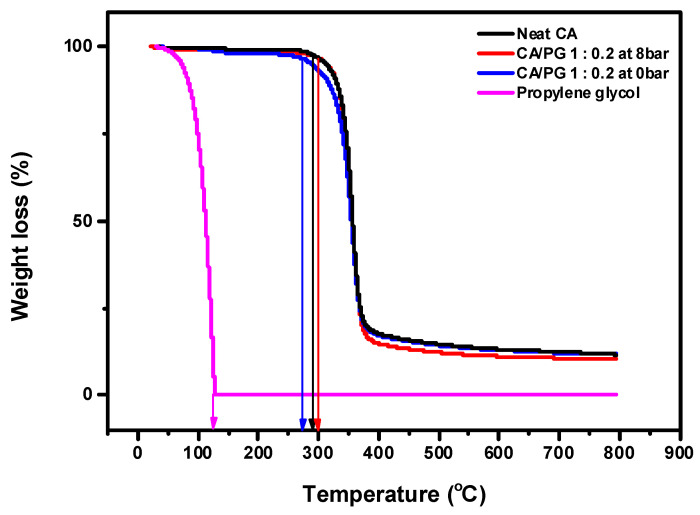
TGA of 1:0.2 CA/propylene glycol (8 bar, 0 bar), neat CA, and propylene glycol.

A TG analysis was carried out to evaluate the thermal stability of the neat CA, 1:0.2 CA/propylene glycol before and after the water pressure treatment and propylene glycol. The neat CA and 1:0.2 CA/propylene glycol (8 bar) were disintegrated at approximately 300 °C, while 1:0.2 CA/propylene glycol (0 bar) was decomposed at approximately 270 °C. A very small weight reducing of 1:0.2 CA/propylene glycol (8 bar) occurred below 100 °C, owing to the small amount of propylene glycol remaining in the polymer regions other than the pores, according to the FTIR spectra. Since the boiling point of propylene glycol is 120 °C, most of the propylene glycol that remains without casing plasticization in the membrane was vaporized at temperatures below 120 °C. The small weight losses of 1:0.2 CA/propylene glycol (8 bar) are attributed to the evaporation of the additive or solvent. The propylene glycol dispersed in the CA polymer occur in the plasticization of the CA chains. Owing to the plasticization, the decomposed temperature of 1:0.2 CA/propylene glycol (0 bar) was approximately 30 °C lower than that of the pure CA. Compared to 1:0.2 CA/propylene glycol (0 bar), in 1:0.2 CA/propylene glycol (8 bar), most of the propylene glycol was released upon the water pressure treatment, such as the neat CA (Figure 4). Although propylene glycol remained in the pore-free portion even after the water pressure treatment (see FTIR spectra), the plasticization was not clearly observed in the TG analysis as the amount of propylene glycol was small. Although the manufactured membrane is a cellulose-based material, it has a high decomposition temperature of 300 °C, so it is confirmed that there is heat resistance.

### 3.4. Porosity of CA/Propylene Glycol

**Figure 5:**
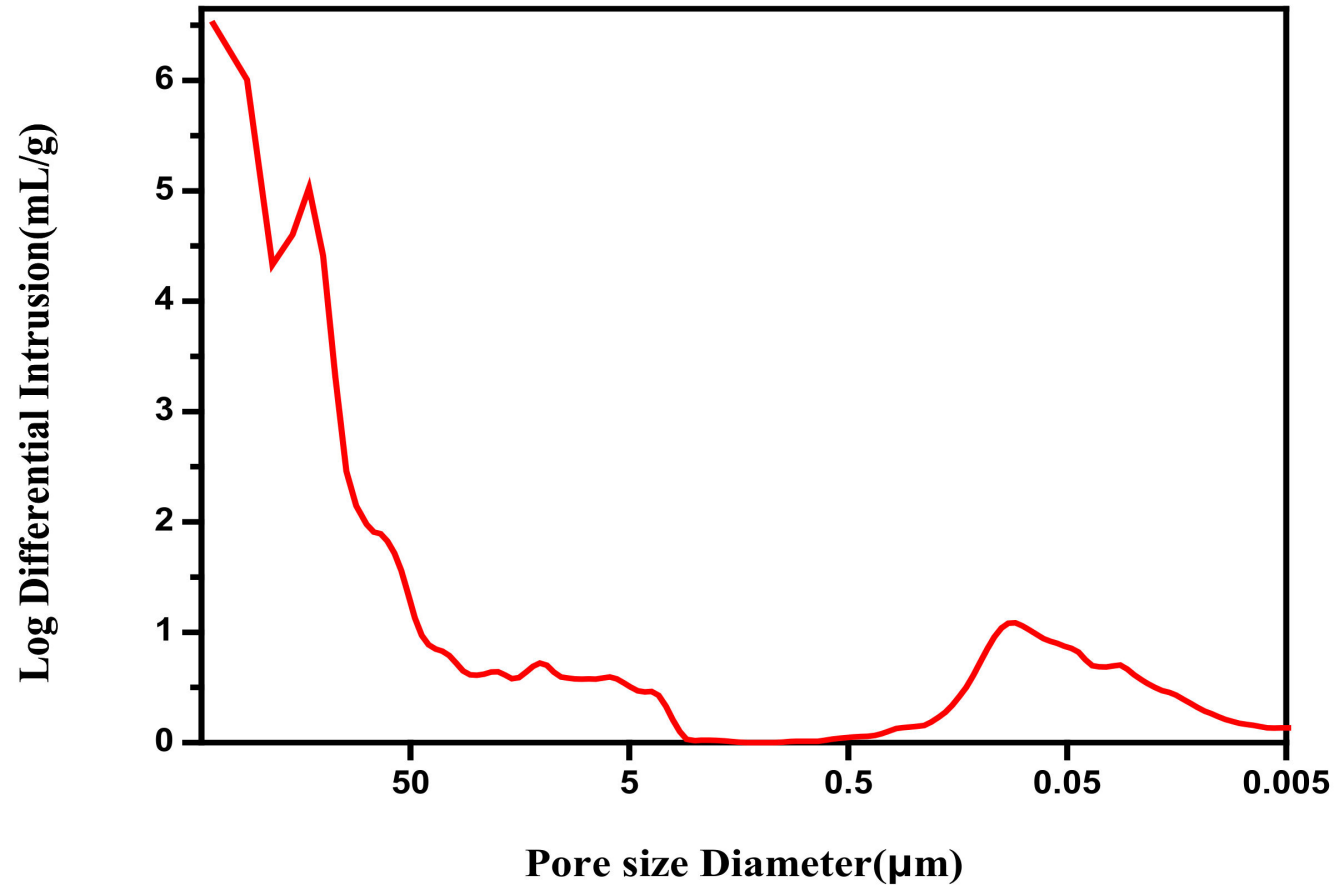
Log differential intrusion of 1:0.2 CA/propylene glycol (8 bar).

The porosity of the CA film was measured using the mercury intrusion method. Figure 5 shows the mercury intrusion according to the pore size. It was confirmed from Table 1 that the bulk density, average pore diameter, and porosity of the 1:0.2 CA/propylene glycol film were 0.13 g/mL, 300 nm, and 69.7%, respectively. Compared to previous studies using glycerin as an additive, the average pore size (300 nm) is smaller, and the pores are evenly distributed. Propylene glycol has fewer hydrophilic groups per molecule than glycerin, resulting in a smaller hydration zone size when dispersed between polymer chains. As the water pressure was applied to the smaller hydration region, the average pore diameter was reduced compared to those in previous studies. In addition, the porosity was reduced compared to those in the previous studies. The CA membrane exhibited a sufficiently high porosity and more compact structure than those in the previous studies to show 13.85% [26]. Thus, it is expected that the pore size and porosity of the membrane can be controlled by the number of hydroxyl groups.

### 3.5. Water Flux Analysis

The pore size of the CA film could be adjusted by the composition of propylene glycol. The water flow of CA/propylene glycol (1:x) (8 bar) increased in the x value from 0.1 to 0.2, and then decreased after 0.4. In the ratio range of 0.1 to 0.4, the propylene glycol additive was evenly dispersed, and the CA was fabricated using the free-standing method. The data of each ratio is the average of 15 or more experiment, and the error from the average is around 20%.

**Figure 6:**
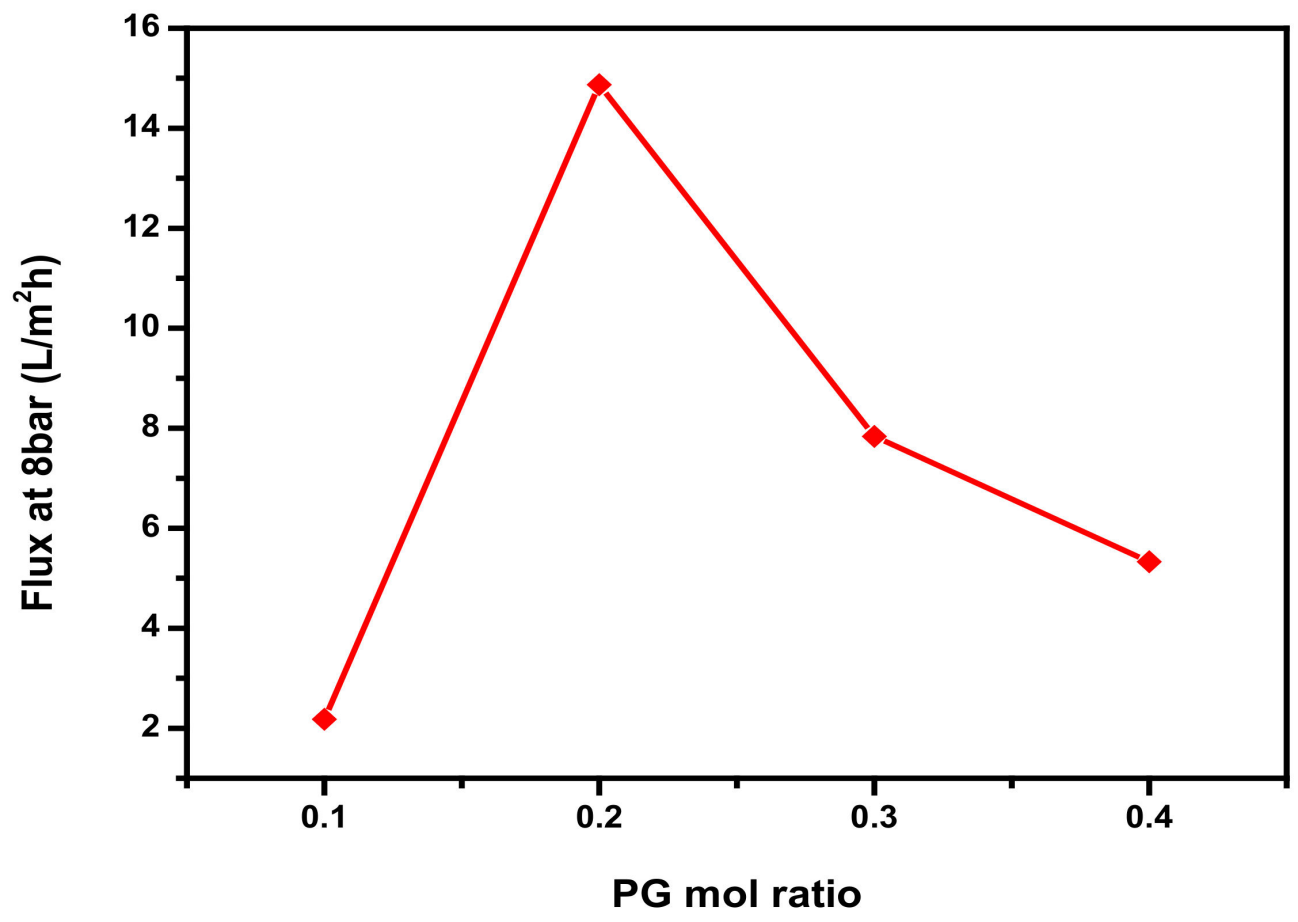
Water fluxes of CA/propylene glycol in the ratio range of 0.1 to 0.4 at 8 bar.

In contrast, at ratios above 0.5, the propylene glycol was not evenly dispersed, and the experiment data were less reproducible. For ratio after 0.5, the experimental error from the average is around 90%. The water flux measurement data measures the amount of water that has passed while opening the curtain. Therefore, there is a lot of excess water to prevent high flux. In other words, there are many pores. Accordingly, the 1:0.2 CA/propylene glycol membrane exhibited the highest water flux (14.87 L/m^2^h), which was the best composition for pore generation in the CA in shown in Figure 6.

## 4. Conclusions

**Figure 7:**
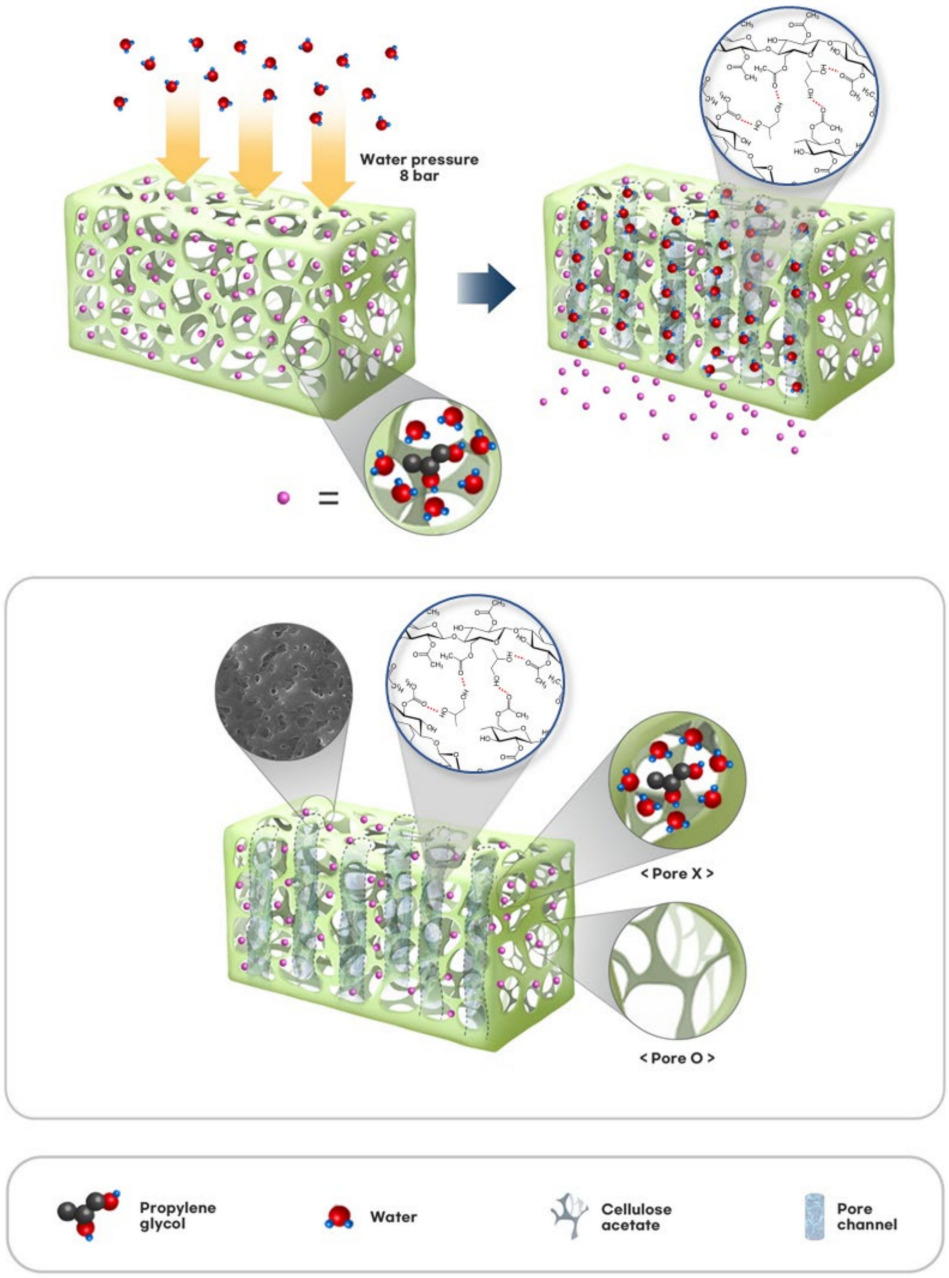
Schematic diagram of plasticized membrane and water treatment process.

In the previous study, we suggested the pore-generation method using a small-molecular weight low-cost organic material without inorganic substance with physical force. In this study, the process was advanced using propylene glycol as an additive. Propylene glycol is eco-friendly, easily controllable, and low-cost. It also provides a strong hydration effect. In addition, the propylene glycol provided a smaller hydrated region than that of glycerin, as the dispersed propylene glycol in the CA polymer had fewer hydroxyl groups than glycerin. Therefore, owing to the small size of the plasticized area, the generated pore-size by the physical force could be controlled more easily by the number of hydroxyl groups, as shown in Figure 7. In particular, as the number of hydroxyl groups increased, the plasticization area became also abundant. In addition, the membrane exhibited a large porosity and small pores owing to the abundant hydration effect of the propylene glycol. Among the results of experiments with various compositions, the maximum flux was 14.87 L/m^2^h. The bottleneck phenomena upon the water pressure treatment were caused by the generated pores in the CA had small sizes. The FTIR spectra was used to confirm the interactions of the propylene glycol with the CA chains before and after the water pressure treatment. The flexibility of the membrane was increased by the remained propylene glycol in the CA matrix. The plasticization of the CA chains by the hydrated propylene glycol was confirmed by the TG analysis. The average pore diameter and porosity were 300 nm and 69.7%, respectively, smaller than those in the previous studies, which use glycerin as an additive. The comparison results are shown in the following Table 2 [28]. Thus, the proposed nanoporous polymer membranes are more flexible and compact than those in the previous study. This mechanism of CA plasticization and water treatment process is shown in Figure 7. Through this study, we sought to confirm how organic substances with hydroxyl groups cause sign hydration effect. However, due to the limitation of too few controls, additional studies are essential for 3 carbon alcohols and di-alcohols of various structure. It is expected that these small pores can be applied to various fields, such as, by bonding this membrane on top of the existing porous membrane to increase the mechanical strength and applying it as a sensing pretreatment filter and separator.

## Figures and Tables

**Table 1:** Porosimetry data of 1:0.2 CA/propylene glycol (8 bar).

Material	Parameter	Value
	Bulk density	0.13 g/mL
Water-pressure-treated CA with propylene glycol	Average pore diameter	300 nm
	Porosity	69.7%

**Table 2:** Porosimetry data according to additive.

Additive	Glycerin	Propylene Glycol
Bulk density	0.14 g/mL	0.13 g/mL
Average pore diameter	630 nm	300 nm
Porosity	78.3%	69.7%

## Data Availability

The data presented in this study are available on request from the corresponding author.
